# Neurogenic organ dysfunction syndrome after acute brain injury

**DOI:** 10.1186/s40779-025-00662-8

**Published:** 2025-11-07

**Authors:** Heng Zhang, Wen-Jin Chen, Yan-Gong Chao, Nan Su, Chiara Robba, Marek Czosnyka, Peter Smielewski, Zofia Czosnyka, Wei He, Xiao Hu, De-Zhong Yao, Cheng-Gong Hu, Min Zhou, Yun-Jie Wang, Xiao-Chun Ma, Xiu-Yun Liu, Dong Ming

**Affiliations:** 1 Department of Neurosurgery, the First Hospital of China Medical University, Shenyang, 110001 China; 2 Department of Neurosurgery, Neuroscience ICU, Xuanwu Hospital Capital Medical University, Beijing, 100032 China; 3 Department of Critical Care Medicine, the First Hospital of Tsinghua University, Beijing, 100016 China; 4 State Key Laboratory of Advanced Medical Materials and Devices, School of Medicine, Tianjin University, Tianjin, 300072 China; 5 IRCCS for Oncology and Neuroscience, San Martino Policlinico Hospital, 16132 Genoa, Italy; 6 Department of Clinical Neurosciences, University of Cambridge, Cambridge, CB1 0QQ UK; 7 Department of Critical Care Medicine, Beijing Tongren Hospital, Capital Medical University, Beijing, 100730 China; 8 Nell Hodgson Woodruff School of Nursing, Emory University, Atlanta, GA 30322 USA; 9 Ministry of Education Key Laboratory for Neuroinformation, School of Life Science and Technology, University of Electronic Science and Technology, Chengdu, 611731 China; 10 Department of Critical Care Medicine, West China Hospital, Sichuan University, Chengdu, 610041 China; 11 Department of Critical Care Medicine, the First Affiliated Hospital of USTC, Division of Life Science and Medicine, University of Science and Technology of China, Hefei, 230001 China; 12 Department of Critical Care Medicine, the First Hospital of China Medical University, Shenyang, 110001 China; 13 Haihe Laboratory of Brain-Computer Interaction and Human-Machine Integration, Tianjin, 300380 China; 14 School of Pharmaceutical Science and Technology, Tianjin University, Tianjin, 300072 China

**Keywords:** Neurogenic organ dysfunction syndrome, Acute brain injury, Hierarchical modular structure, Hierarchical hub, Small-world network, Systemic homeostasis

## Abstract

Systemic complications are common after acute brain injury (ABI) and may trigger coagulation cascades, systemic inflammation, as well as dysfunction of the cardiovascular, respiratory, and gastrointestinal systems, etc. The pathogenesis of these systemic manifestations is multifactorial but not yet fully elucidated. This paper introduces the novel term neurogenic organ dysfunction syndrome (NODS) to characterize systemic instability arising from internal and external perturbations of the neuronal center following ABI. Elucidating the central neurogenic mechanisms of NODS is critical for early detection and prevention of complications, thereby reducing mortality and improving patient outcomes following ABI. In this paper, we explore the potential central neurogenic mechanisms of NODS from the perspective of complex brain network theory, focusing on the structural network of the central autonomic system (CAS) that maintains systemic stability, and the functional network governed by the central stress system (CSS). The CAS can be divided into the cortical autonomic network, which involves higher cortical regions, and the subcortical autonomic network, which is relatively conserved, with its main connections located in deep brain structures. The CSS is a large-scale complex network characterized by hierarchy, hubs, and modularity, which together enable the competitive optimization of functional segregation and integration. Under physiological conditions, modules (mediating functional segregation) and hubs (functional integration) within the CSS dynamically trade-off with each other to maintain the overall homeostasis. However, this balance is disrupted following pathological insults or injury, resulting in weakened functional integrity of the CSS following ABI, impaired module activity, and disturbed hub integration. This paper also demonstrates the distinct pathological manifestations arising from disturbances at different levels of the homeostatic system. Finally, this study proposes potential clinical interventions, including analgesia and sedation, neuromodulation, and receptor regulation, for early interventions and potential treatment of NODS, aiming to improve patient outcomes.

**Supplementary Information:**

The online version contains supplementary material available at 10.1186/s40779-025-00662-8.

## Background

**Fig. 1:**
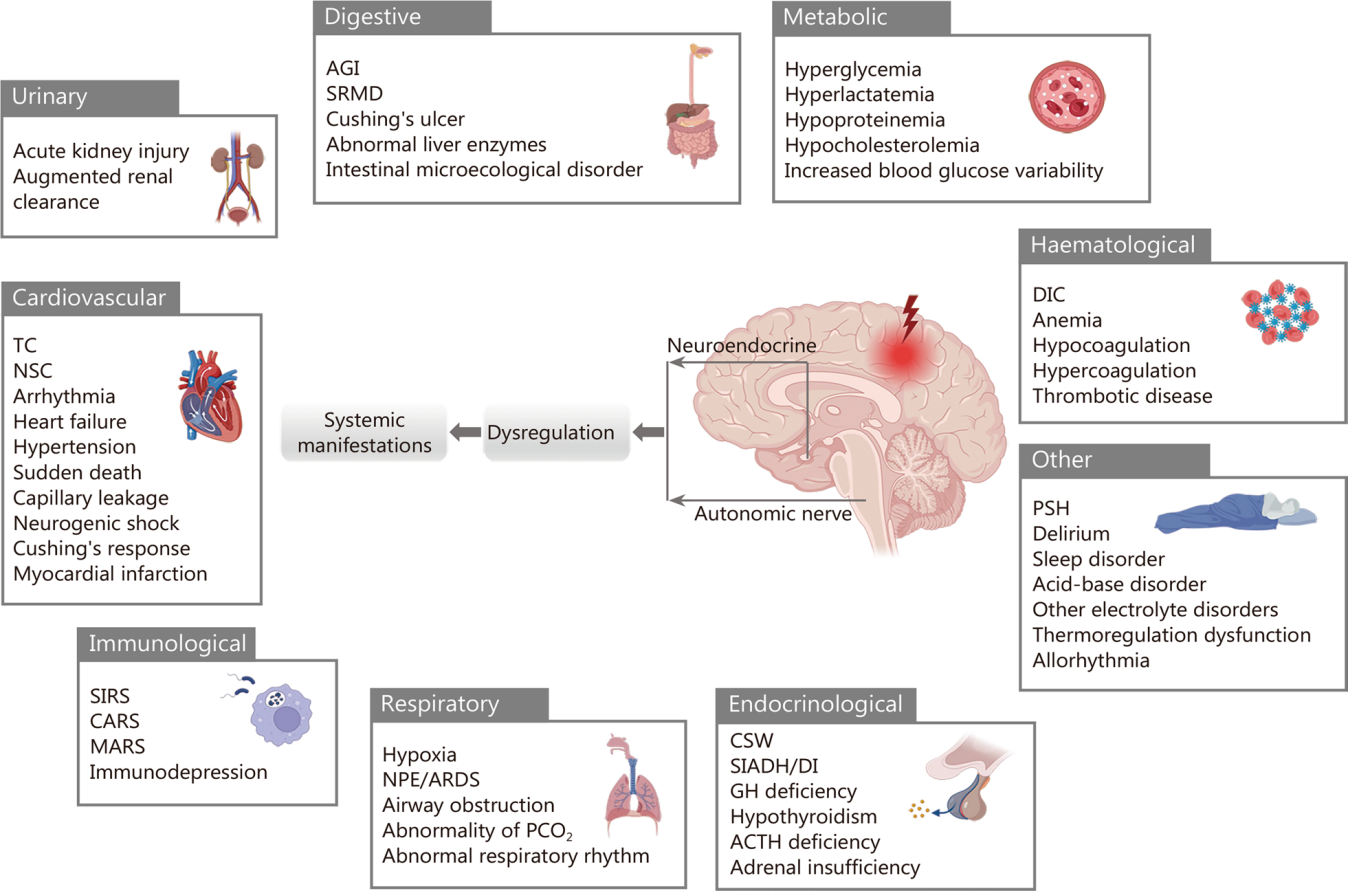
ABI-induced neurogenic organ dysfunction syndrome (NODS). After ABI, signals are transmitted to peripheral organs through the efferent segment of the autonomic nervous system (ANS) and neuroendocrine pathways, causing structural changes and/or functional damage to multiple subsystems or organs, resulting in multisystem complications. Arrows indicate that dysregulated signals are transmitted via neuroendocrine and autonomic pathways, leading to systemic manifestations. TC takotsubo cardiomyopathy, NSC neurogenic stress cardiomyopathy, SIRS systemic inflammatory response syndrome, CARS compensatory anti-inflammatory response syndrome, MARS mixed antagonist response syndrome, NPE neurogenic pulmonary edema, ARDS acute respiratory distress syndrome, PCO_2_ partial pressure of carbon dioxide, PSH paroxysmal sympathetic hyperexcitation, AGI acute gastrointestinal injury, SRMD stress-related mucosal disease, CSW cerebral salt wasting, SIADH syndrome of inappropriate antidiuretic hormone secretion, DI diabetes insipidus, GH growth hormone, ACTH adrenocorticotropic hormone, DIC disseminated intravascular coagulation

Acute brain injury (ABI), including traumatic brain injury (TBI), subarachnoid hemorrhage, and stroke, is a major cause of mortality and poor neurological outcomes [1]. The incidence of ABI has increased dramatically, and patients with ABI often require prolonged hospital stays and intensive care unit (ICU) management. Although numerous studies have investigated the mechanisms of ABI, research on its effects on extracranial organ dysfunction remains limited. The concept dates back over a century, when Cushing’s ulcers and reflexes were first raised [2, 3]. In recent years, there has been a growing recognition that systemic multi-system/organ complications following ABI constitute a set of clinically observable phenomena that can be repeatedly observed, such as neurogenic stress cardiomyopathy [4], neurogenic pulmonary edema (NPE) [5], paroxysmal sympathetic hyperexcitation (PSH) [6–8], cerebral salt wasting (CSW) [9, 10], syndrome of inappropriate secretion of antidiuretic hormone [11]. Systemic complications following ABI also include delirium [12], post-traumatic stress disorder [13, 14], as well as other psychological and neurological disorders [15, 16]. The pathophysiology of multiple organ dysfunction syndrome (MODS) is traditionally attributed to humoral regulatory mechanisms, such as systemic inflammatory response syndrome or compensatory anti-inflammatory response syndrome. MODS is a critical illness characterized by reversible physiological abnormalities involving dysfunction of two or more organs simultaneously, leading to longer ICU stays and higher mortality (27 – 100%) [17]. MODS occurs frequently following ABI and plays an important role in patient outcomes [16, 18]. Åkerlund et al. [19] reported worse outcomes in patients with moderate ABI and concurrent metabolic disorders, compared with patients with severe ABI but normal metabolism. Consequences of MODS following ABI may include sympathetic system dysfunction, excessive proinflammatory response, disturbed metabolism, reduced cerebral blood flow, hypoxia, acidosis, and bleeding, collectively contributing to homeostatic destabilization [20–22]. This paper introduces the novel term neurogenic organ dysfunction syndrome (NODS) to characterize systemic instability arising from internal and external perturbations of the neuronal center following ABI (Fig. 1 and Additional file 1: Table S1).

Identifying the fundamental mechanisms of NODS is crucial for reducing mortality and developing early interventions. With the progress of neuroscience, research has demonstrated that the central nervous system (CNS) serves as the primary control center for maintaining systemic stability, including the respiratory system [23], circulatory system [24], and basic homeostasis [25–28]. However, most studies have mainly focused on individual organs rather than systemic dysfunction [23–28]. Moreover, the role of the injured brain itself in organ dysfunction, independent of the effects of concurrent polytrauma, remains poorly understood. To address these issues, we adopt the basic concept of complex brain networks[29], including structural brain networks (physical, anatomical connections) and functional brain networks (patterns of neural activity) [30], as well as modularity (the organization of nodes into modules with dense internal connections) [30, 31], hubs (nodes with high centrality) [32, 33], and hierarchy (functional implications including fractal subdivision, hub integration span, and information processing order) [33, 34], to explain the physiological mechanisms by which the CNS maintains systemic homeostasis. We also apply it to further explain the clinical manifestations of NODS and to clinical interventions for its early treatment, to improve patient outcomes.

## The control center that maintains systemic stability

Spontaneous homeostasis is the most fundamental condition for sustaining human life [35, 36], which involves numerous biological parameters, including respiration, blood circulation, body temperature, blood gas, glucose, osmolality, and inflammation. Two terms are commonly used to describe systemic stability: homeostasis and allostasis [37–40], both of which consider the brain as the control center of systemic stability, viewed from the perspectives of feedback and feed-forward theory [41–43]. The homeostasis control system consists of 2 networks: a structural network based on the central autonomic system (CAS) and a functional network governed by the central stress system (CSS).

### The structural brain network for homeostasis maintenance

**Fig. 2:**
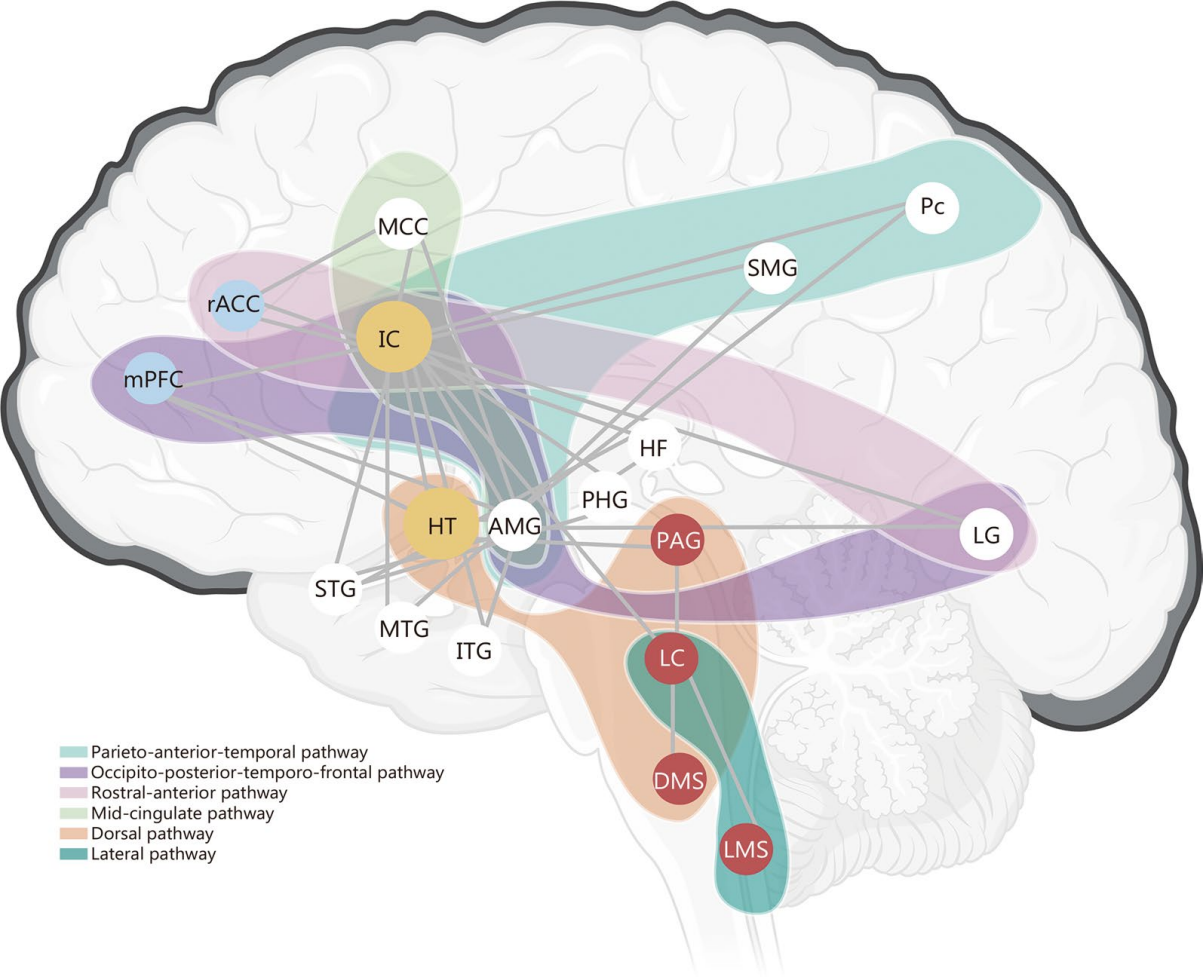
Schematic diagram of the central autonomic system (CAS). The CAS comprises the cortical autonomic network (CAN) and the subcortical autonomic network (SAN), forming the structural network that serves as the control center for systemic stability. Higher-order cortical regions, including the medial prefrontal cortex (mPFC), insular cortex (IC), and cingulate cortex, are involved in CAN and are connected via 3 white matter pathways: the parieto-anterior-temporal, the occipito-posterior-temporo-frontal, and the limbic pathways (divided into a rostral-anterior pathway and a mid-cingulate pathway). Network nodes of SAN include hypothalamus (HT), periaqueductal gray (PAG), locus coeruleus (LC), dorsal medullary seed (DMS), and lateral medullary seed (LMS). White matter pathways in SAN are divided into dorsal and lateral pathways, with the insula and HT acting as connector hubs between SAN and CAN. MCC mid cingulate cortex, rACC rostral anterior-cingulate cortex, HF hippocampal formation, PHG parahippocampal gyrus, SMG supramarginal gyrus, Pc precuneus, AMG amygdala, LG lingual gyrus, STG superior temporal gyrus, MTG middle temporal gyrus, ITG inferior temporal gyrus

Several structural network models have been proposed regarding homeostasis maintenance, including the central autonomic network [44, 45], the neurovisceral integration model [46, 47], the polyvagal theory [48], the structural connectome of the central homeostatic network [49], and the cortical autonomic neural network [50]. Reisert et al. [51] provided a global view of the CAS and reconstructed the structural network underlying the ANS using diffusion-weighted imaging from the Human Connectome Project database. The CAS, a representative structural brain network responsible for maintaining homeostasis, can be divided into the cortical autonomic network (CAN) and the subcortical autonomic network (SAN) (Fig. 2).

The CAN involves several higher cortical regions, including the medial prefrontal cortex, insular cortex, and cingulate cortex [50]. Three white matter pathways also play critical roles in CAN: the parieto-anterior-temporal pathway, the occipito-posterior-temporo-frontal pathway, and the limbic pathway (divided into a rostral-anterior pathway and a mid-cingulate pathway) [51]. In contrast to the CAN, the SAN is relatively conserved, with its primary connections located in deep brain structures, including the hypothalamus, periaqueductal gray (PAG), locus coeruleus (LC), dorsal medullary seed, and lateral medullary seed. The main white matter pathways of SAN consist of dorsal and lateral pathways. In addition, the insula and hypothalamus act as the connector hubs between CAN and SAN [51–53].

### The functional brain network for homeostasis maintenance

**Fig. 3:**
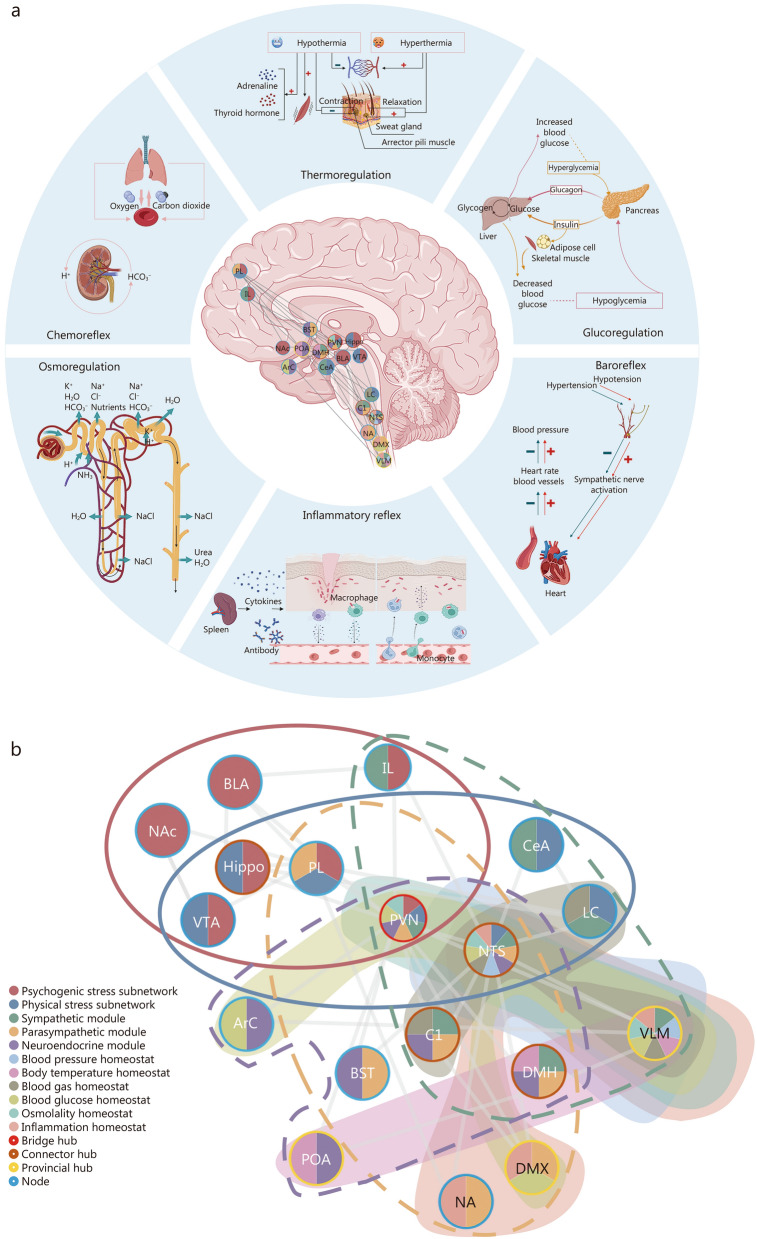
Schematic diagram of the central stress system (CSS). **a** The relative anatomical location of the hubs in the brain, including those involved in thermoregulation, glucoregulation, chemoreflex, osmoregulation, baroreflex, and inflammatory reflex. **b** The hierarchical module network is represented by a coil, and the highest module (solid line) includes the psychological and physical stress subnetworks. The secondary modules (dashed lines) include sympathetic, parasympathetic, and neuroendocrine modules. The lower modules (filled areas) encompass homeostats necessary for maintaining homeostasis, including, but not limited to, homeostats regulating body temperature, blood gas, blood glucose, blood pressure, osmolality, and inflammation. Hub levels are represented by circles, and different border colors represent bridges, connectors, provincial hubs, and ordinary nodes, as shown in the figure legend. Provincial hubs exhibit high intra-module connectivity, connector hubs have high inter-module connectivity, and bridge hubs represent global integration. PVN paraventricular nucleus of the hypothalamus, NTS nuclei of the tractus solitarius, DMX dorsal motor nucleus of the vagus nerve, LC locus coeruleus, VLM ventrolateral medulla, IL infralimbic cortex, PL prelimbic cortex, CeA central amygdala, DMH dorsomedial hypothalamus, NA nucleus ambiguus, BST bed nucleus of the stria terminalis, VTA ventral tegmental area, NAc nucleus accumbens, Hippo hippocampus, BLA basolateral nucleus, C1 C1 neurons, POA preoptic area, Arc arcuate nucleus

The functional brain network refers to the connections between individual neurons and neuron clusters that are engaged during the execution of specific tasks, which normally involve dynamic interactions and coordination among multiple brain regions [30, 54]. Different functional brain networks have been proposed to support homeostasis maintenance [55–58], with the CSS being the most representative, specialized in processing information related to internal and external sensations as well as visceral movement [59, 60]. In much of the literature, the stress system is generally regarded as a key entity for homeostasis maintenance and for inducing peripheral responses [36, 61–63]. However, the understanding of how the central components of the stress system perform neural information processing and operation remains limited to simple descriptions. We attempt to apply the theoretical framework of complex brain networks to reorganize relevant neurophysiological materials and describe the central neurogenic mechanisms underlying homeostasis maintenance. Figure 3a illustrates the relative anatomical locations of hubs in the brain, including those governing thermoregulation, glucoregulation, chemoreflex, osmoregulation, baroreflex, and inflammatory reflex. These hubs form the basis for basic homeostatic regulation within the CSS. As shown in Fig. 3b, the CSS forms a large-scale complex network with small-world, hierarchical hubs and hierarchical modules, which collectively support this highly-efficient massive system and ensure the competitive optimization of information segregation and integration [64–67].

#### Hierarchical modular structure: the separated network supports efficiency for specific brain functions

Modularity is an essential biomarker of brain networks associated with efficient and complex brain functions. It refers to individual brain subnetworks that are segregated from other modules within the whole-brain network, with networks exhibiting high modularity having dense intra-module connections and sparser inter-module connections [68, 69]. Modularity can be characterized using graph theory, in which large-scale brain networks are comprised of individual brain regions (nodes) and the connections between them (edges). Complex brain functions require multiple modules to execute regional and specific functions efficiently. These modules often exhibit scale-free or hierarchical properties, whereby smaller modules can be embedded within larger ones and perform more specialized functions [31, 33, 64, 70–74].

The CSS functions as a representative hierarchical modular network, integrating multiple brain networks, and can be divided into three tiers. Tier-1 is composed of sub-networks mediating physical stress and psychological stress [75]. Tier-2 includes brain regions and nuclei responsible for sympathetic, parasympathetic, and neuroendocrine functions [76, 77]. Tier-3 consists of homeostats essential for maintaining homeostasis, such as regulators of temperature, blood gas, inflammation, blood pressure, blood glucose, and osmolality [25, 27, 28, 61, 77–81]. Several Tier-3 modules may share the same nuclei. For example, the nuclei of the tractus solitarius (NTS), C1 neurons, and LC play important roles in maintaining blood gas homeostasis, which also regulate respiration [23, 78]. Some Tier-3 modules can be further subdivided, such as the osmolality module, which comprises water balance and sodium balance sub-modules [82]. This hierarchical modularity network enables the CSS to achieve a stepwise functional specialization from central to peripheral levels. Psychological or physical stressors trigger the corresponding sub-networks, eliciting a series of responses, including activation of the sympathetic nervous system (SNS) and hypothalamus–pituitary–adrenal axis, as well as suppression of the parasympathetic nervous system. At the Tier-3, dynamic changes occur in the cardiovascular, respiratory, metabolic, and immune systems, ultimately extending to peripheral target organs under the regulation of the nervous system and chemical neurotransmitters.

#### Hierarchical hub: the hub connecting the internal and external worlds

Hubs are nodes with high centrality, occupying central positions in both structural and functional brain networks [30, 33, 83]. They can be further classified as provincial hubs and connector hubs [65]. Provincial hubs mainly connect nodes within the same functional module, whereas connector hubs mainly link nodes across different modules [67]. Hierarchical hubs integrate information across levels, from global and inter-module networks to intra-module networks. From the perspective of neurophysiology and functional anatomy, hierarchical hubs enable the CSS to establish distributed and interactive connections from the cortex to the brainstem, integrating different modules into a functional integrity. The fundamental survival modules of the CSS are usually regulated by provincial hubs. As shown in Fig. 3, the preoptic area primarily regulates body temperature homeostasis, while the ventrolateral medulla regulates blood pressure [25, 61]. As connector hubs, the paraventricular hypothalamic nucleus (PVN) and the nucleus of the NTS integrate the sympathetic and parasympathetic systems at the subcortical and brainstem levels, respectively [84, 85]. The prefrontal cortex, hippocampus (Hippo), PVN, and ventral tegmental area also act jointly as connector hubs, managing physical and psychological stress [75]. A study of chronic moderate-to-severe TBI found that patients with depressive symptoms showed significantly enhanced resting-state functional connectivity between the anterior prefrontal cortex and multiple sensory and motor cortical regions [86]. A specific type of connector hub, termed a bridge hub, refers to nodes that strongly connect inter-modules. It plays a critical role in efficient global communication [65]. In the CSS, the PVN has the most overlapping connections between modules, managing both psychological and physical stress, integrates the ANS and neuroendocrine system [76, 87], and is also involved in the regulation of osmolality and blood glucose [27, 28, 61, 80]. A study demonstrated that mild blast TBI induces persistent hyperactivity in corticotropin-releasing factor neurons within the PVN, resulting from dysfunction of gamma-aminobutyric acid-ergic (GABAergic) synapses [88]. Perturbation of the PVN increases the network vulnerability of the CSS. Due to the “embedded” nature of hierarchical modularity, the CSS only integrates part of the hubs across different hierarchical modules.

#### Small-world network: the guarantee for achieving the optimal global efficiency

**Fig. 4:**
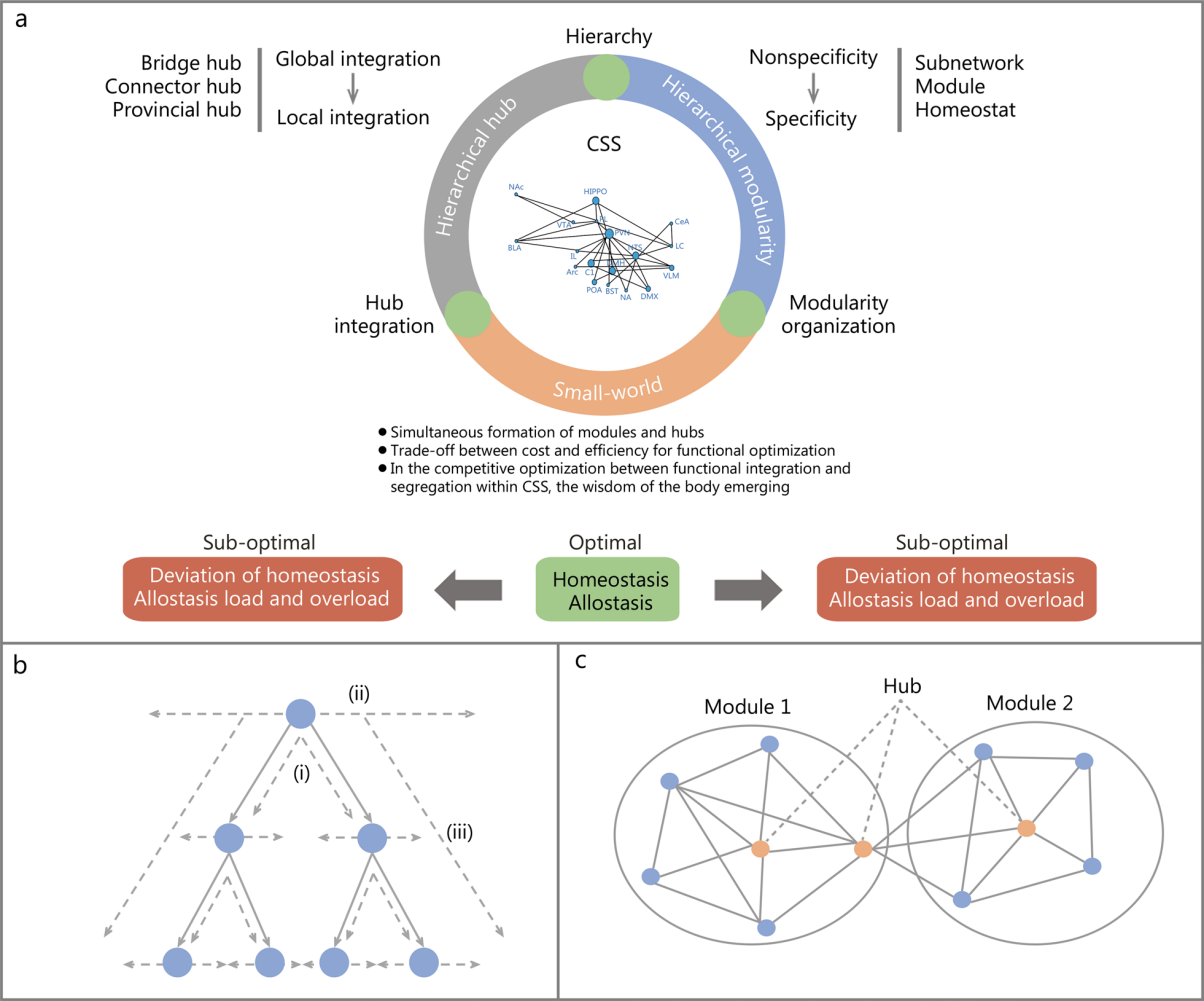
Schematic diagram of the physiological and pathophysiological network operation mechanisms of the central stress system (CSS). **a** According to the basic organizational principles of functional brain networks, the CSS exhibits hierarchical, modular, and hub-integration properties, which interact through hierarchical modularity, hierarchical hub integration, and small-world networks to execute the neural information processing. Hierarchical modularity organizes CSS modules in a fractal manner, enabling stepwise subdivision and specialization from central to peripheral levels. Hierarchical hub integration enables the integration of hubs within CSS across global, inter-module, and intra-module levels according to their integration span, thereby achieving functional integration. The small-world network organization balances the trade-off between the cost and efficiency imposed by the former two networks, optimizing the segregation and integration of CSS functions and giving rise to the “wisdom” that maintains systemic homeostasis[94]. Under physiological or psychological stress, a series of acute or chronic stress-related diseases may occur when the network organization of the CSS is suboptimal, even in the absence of structural changes. Disruption of the small-world networks can shift brain function from an optimal state to a sub-optimal state. **b** Hierarchy includes the following functional implications: (i) “fractal” refers to the repeated encapsulation of smaller modules within larger ones, with the hierarchy of this type of (dys)function reflected in the degree of functional subdivision; (ii) “reach” refers to the integration span of hubs, with the hierarchy of such (dys)function manifested in the extent of function integration; (iii) “ordering” refers to the sequence of information processing, with the hierarchy of this type of (dys)function expressed as a top-to-bottom chain of command. **c** Modularity organization, and hub integration. A module is a group of nodes that have dense intra-module connections and sparse connections with nodes outside the module. A hub is a node with a high degree or high centrality. PVN paraventricular nucleus of the hypothalamus, NTS nuclei of the tractus solitarius, DMX dorsal motor nucleus of the vagus nerve, LC locus coeruleus, VLM ventrolateral medulla, IL infralimbic cortex, PL prelimbic cortex, CeA central amygdala, DMH dorsomedial hypothalamus, NA nucleus ambiguus, BST bed nucleus of the stria terminalis, VTA ventral tegmental area, NAc nucleus accumbens, Hippo hippocampus, BLA basolateral nucleus, C1 C1 neurons, POA preoptic area, Arc arcuate nucleus

The human brain network exhibits the topological characteristics of the small-world networks, quantified by a higher local clustering coefficient and a relatively short path length between distant nodes [89–91]. Real complex brain networks lie between random and regular networks [33, 72], achieving optimal efficiency via trade-offs between modules and hubs [70, 92–94]. This small-world network topology endows the brain with emergent functional properties [90]. Therefore, the small-world networks serve as one of the primary structural bases for competitively optimizing wiring cost and communication efficiency in the structural networks, as well as segregation and integration in the functional networks [95–98]. The above-mentioned hierarchical modularity structure supports functional segregation from the center to the periphery, while hierarchical hubs facilitate integration from localized to global functional coordination in the control center. Over millions of years of evolution, the function of the control center has been optimized, giving rise to sophisticated mechanisms that maintain systemic stability. As shown in Fig. 4a, based on the nature of small-world network topology, the CSS exhibits a distribution from optimal to sub-optimal in terms of functional segregation and integration for maintaining homeostasis. Especially when it shifts to a sub-optimal state under internal and external perturbations, the whole-body manifests deviation of homeostasis or allostasis load and overload caused by modular organization and/or hub integration. Such disruption may lead to a series of diseases associated with impaired modular organization and/or hub integration [99].

## Possible pathophysiological mechanisms of ABI-induced NODS

Whole-body homeostasis is maintained by the systemic organization and operation of the CNS network. Following ABI, any damage to the brain may compromise structural or functional integrity, disrupt relevant brain functions, and ultimately lead to NODS (Fig. 4).

### Modularity and NODS

ABI-induced impairment of hierarchical brain network modularity is associated with highly localized network alterations, often confined to the local modules of the functional network, with the hierarchy of the affected modules determining the clinical manifestations [99]. In cases of impaired cardiovascular function following ABI, the clinical manifestations depend on the tier of the perturbed module [100–102].

If the physical or psychological stress sub-network is disturbed, a systemic stress response will occur, known as the general adaptation syndrome [103, 104]. Perturbation of the Tier-2 module, such as the SNS, can result in sympathetic excitation of the heart, including increased heart rate and blood pressure, as well as systemic effects of sympathetic innervation on other effectors [22, 105–108]. Heart rate variability is lower in individuals who experienced moderate to severe ABI compared with controls, indicating increased SNS activity and decreased parasympathetic nervous system function following ABI, which serves as clear evidence of autonomic dysfunction [109]. Disturbance of the Tier-3 module, such as the cardiovascular center, primarily affects the ANS and sympathoadrenal system [4, 110], with minimal impact on other systems. Moreover, disruption of the cardiac sympathetic and sympathoadrenal modules can lead to abnormalities in the heart base or apex, respectively [100]. Therefore, by analyzing the impaired peripheral dysfunction, it is possible to infer the disruption of central modules caused by ABI, helping to distinguish stress cardiomyopathy from neurogenic stress cardiomyopathy in the above cases [101, 102].

### Hub integration and NODS

In ABI, disturbances to the functional or structural brain hubs may differentially affect functional integration within and between modules [111]. Damage to central hubs represents a hotspot in unconscious patients after brain injury [112], impacting global, inter-module, or even intra-module integration [99]. Moreover, in most PSH cases, although structural disturbances occur in various brain regions, stimulation or disinhibition of PAG may lead to similar clinical manifestations, such as abnormalities in the coordinated functions of sympathetic, motor, and pain-modulating neural circuits [6]. Clinical reports have also confirmed that corpus callosum, medial temporal lobe, and diencephalo-mesencephalic injury increase the risk of PSH [7, 113, 114], due to the disinhibition of the PAG. These findings suggest that the PAG may be one of the brainstem hubs in this pathophysiological process [6].

### Hierarchy and NODS

In this paper, hierarchy includes three functional implications (Fig. 4b). The hierarchy of functional disorders is also reflected in information processing sequences. For example, different levels of brain injury may lead to different patterns of respiratory dysfunction [78]. Specifically, the Cheyne-Stokes respiration is primarily caused by bilateral hemispheric or diencephalic damage, whereas Apneustic breathing is considered a consequence of injury at the level of the Kölliker-Fuse nucleus [115, 116]. Biot’s breathing pattern occurs after medulla oblongata injury, and respiratory arrest arises from damage to the pre-Botzinger complex or upper spinal cord [117].

### Small-world network and NODS

During the dynamic response of the brain network to internal and external perturbations, the small-world network may be sub-optimal, causing the system to gradually shift from allostasis or homeostasis toward allostatic load, overload, or deviation of homeostasis, even in the absence of structural damage to the CAS following ABI. Systemic regulation of the ANS and hypothalamus–pituitary–adrenal axes is mediated by autonomic nervous and neuroendocrine modules and hubs within the CSS, including the hypothalamus, Hippo, and NTS.

Under physiological conditions, modules (mediating functional segregation) and hubs (mediating functional integration) within the CSS dynamically trade-off with each other to maintain the overall homeostasis[94]. However, under pathological or injury conditions, this optimal state is disrupted, leading to abnormal module function or disturbed hub integration. The functional integrity of the CSS is weakened following ABI, manifesting as abnormal function of modules (sympathetic, parasympathetic, and neuroendocrine) and disturbed integration of hubs (PVN and PAG). Specifically, the primary insult and subsequent secondary injury cascades, including neuroinflammation and oxidative stress, lead to neuronal death and synaptic dysfunction within critical hub regions such as the PVN and PAG. Dysregulation of the ANS or neuroendocrine system contributes to clinical manifestations such as inflammatory responses, gastrointestinal ischemia, stress cardiomyopathy, cardiac arrest, CSW, and syndrome of inappropriate antidiuretic hormone secretion. For example, neurogenic hypotension arises from abnormal module function: when the sympathetic module is impaired or the parasympathetic module is overactivated, loss of vasomotor tone and cardiac regulatory control leads to hypotension. Impaired hub integration reduces systemic regulatory efficiency, leading to conditions such as immunosuppression, metabolic syndrome, PSH, and NPE. In conclusion, when the small-world organization shifts toward a suboptimal state following ABI, systemic homeostasis is disturbed either due to module dysfunction or hub impairment.

## Discussion

It is evident that the pathophysiological mechanisms of NODS following ABI involve interactions between the peripheral and central systems, as well as among components within the central system; this paper, however, focuses on the latter.

The complex brain network theory is applied to integrate the fragmented neurophysiological evidence. At the physiological level, this paper aims to address the question of the origin of the body’s “wisdom” for maintaining homeostasis. At the pathophysiological level, this paper attempts to explain how NODS arises from central neurogenic mechanisms.

NODS following ABI is a complex, multi-factorial pathophysiological process [118, 119]. Following ABI, PVN, and PAG exhibit high vulnerability. These nodes serve as convergence points of numerous neural pathways, making them highly susceptible to injury and capable of amplifying local damage in a cascading effect on multiple downstream systems they regulate. In the brain, pathophysiological alterations arising from damage to control centers, including direct mechanical damage, blood–brain barrier disruption, excitotoxicity, oxygen stress, cell death, and mitochondrial dysfunction, disturb systemic homeostasis [120, 121]. Recent evidence also indicates that ABI-induced changes in the immune system, including immune suppression and inflammatory activation, have a profound impact on the brain and the peripheral organs [122–125]. However, the fundamental organization and operation principles of the control centers that regulate systemic homeostasis remain incompletely understood, and advancing this knowledge will potentially fill the gaps in our current understanding of NODS following ABI.

Various terms have been used to describe systemic manifestations following ABI, such as non-neurologic organ dysfunction [126–128], extracranial complications [129, 130], and non-neurological complications [131–133]. In cases of isolated ABI, systemic manifestations can still be observed even when primary systemic inflammatory response syndrome or compensatory anti-inflammatory response syndrome due to infection, pancreatitis, and multiple traumas are excluded. Clinically, it is necessary to consider the potential involvement of central neurogenic mechanisms following ABI. In addition, in MODS caused by other reasons, stress response or secondary brain injury often occur during critical illness, and attention should be paid to the role of central neurogenic mechanisms in this vicious circle.

NODS are not only a stress-related organ dysfunction. First, the classic stress response cannot explain all systemic manifestations following ABI, such as Cushing’s ulcer [134], CSW [9, 10], PSH [6], and NPE [135]. Second, ABI-induced NODS involves the structural and functional integrity of the control center responsible for maintaining systemic stability. Therefore, it is necessary to provide broader and profound explanations from the perspective of complex brain network theory to elucidate the fundamental mechanisms underlying stress-related organ dysfunction.

NODS is a complex system, and its mechanisms cannot be fully explained from a unidimensional perspective [118]. We must consider not only the structure and function of the CNS, but also study the interactions among different modules and hubs.

In clinical investigations of systemic manifestations following ABI, the multiple organ dysfunction score and Sequential Organ Failure Assessment (SOFA) score are used to assess the severity of systemic organ dysfunction caused by ABI. However, it is important to adjust these scores by excluding organs with low incidence, such as the liver, as well as the nervous system [20, 126, 127]. Krishnamoorthy et al. [20] reported a significant difference in prognosis between modified SOFA scores of 0—7 versus ≥ 8 in TBI patients. These classical scoring systems focus on major organs and can partially reflect deviation of systemic homeostasis. However, given the CNS’s role in neurogenic regulation of systemic homeostasis, it is necessary to develop a scoring system that comprehensively reflects systemic stability, enabling accurate assessment of deviation of systemic homeostasis following ABI [136].

**Fig. 5:**
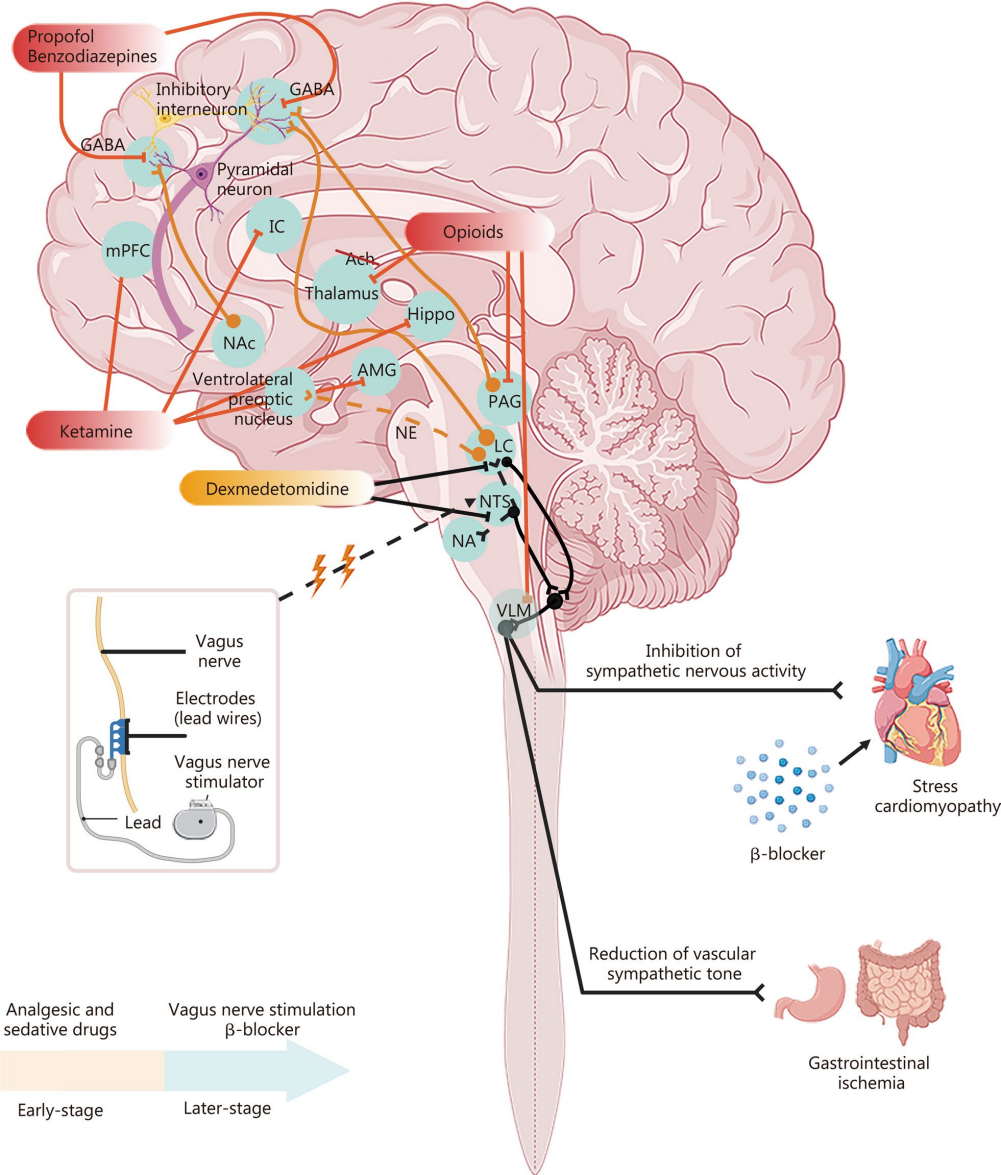
Schematic diagram of early sedation-analgesia, late neuromodulation, receptor regulation, and the roles of the central autonomic system and central stress system. In the early stage after ABI, based on the pharmacological mechanisms of analgesic and sedative drugs on different nodes within the central stress system, these drugs can be used to maintain systemic homeostasis. Dexmedetomidine activates receptors on LC neurons, enhancing inhibitory activity and thereby producing sedation and sympathetic inhibition. It also activates receptors on NTS neurons, further inhibiting sympathetic output while enhancing vagal activity. Together, LC inhibition and NTS activation lead to the inhibition of the ventrolateral medulla (VLM), weakening the pathway of excessive sympathetic output and providing cardioprotection. Dexmedetomidine activates receptors on LC and NTS, which results in reduced excitatory drive to VLM, decreased efferent sympathetic output, lowered sympathetic tone in mesenteric blood vessels, vasodilation, and the restoration and improvement of blood flow. In stress-induced heart disease treated with vagus nerve stimulation, NTS, upon receiving signals, activates NA and enhances its firing, thereby slowing down the heart rate. Additionally, β-blockers exert therapeutic effects in stress-induced heart disease. GABA gamma-aminobutyric acid, mPFC medial prefrontal cortex, IC insular cortex, NAc nucleus accumbens, Ach acetylcholine, Hippo hippocampus, AMG amygdala, PAG periaqueductal gray, NTS nuclei of the tractus solitarius, NA nucleus ambiguous, NE norepinephrine, LC locus coeruleus

Finally, based on the neuropathophysiological mechanisms of NODS following ABI, multiple clinical intervention strategies have been proposed. Targeted analgesia and sedation under neuromonitoring may represent potential approaches for the treatment and prevention of ABI-induced NODS. While ensuring the efficacy and safety of intracalvarium under neuromonitoring, analgesic and sedative drugs can be titrated according to systemic stability targets and regulated variables. In addition to being standard treatment for ABI, these interventions also provide neuroprotective effects and can regulate the excitability and neurotransmission of major nuclei within the CSS. Based on the neuropharmacological mechanisms—with propofol and benzodiazepines acting on GABAergic neurons, ketamine acting on glutamatergic neurons, opioids acting on peptidergic neurons, and dexmedetomidine regulating the excitability of adrenergic neurons—these agents can interfere with the dynamics of brain networks [137–141]. Specifically, as shown in Fig. 5, propofol binds to postsynaptic membranes, enhances GABAergic inhibitory effects, counteracts arousal input signals of pyramidal cells, and reduces their excitatory activity, thereby promoting loss of consciousness. Benzodiazepines enhance the neuroinhibitory effects of GABA. Dexmedetomidine binds to receptors on LC neurons and inhibits norepinephrine release in the ventrolateral preoptic nucleus (VLPO) (inhibition indicated by orange dashed lines). Consequently, the VLPO becomes disinhibited and lowers arousal through its inhibitory output. Opioids bind to receptors in the PAG and ventrolateral medulla, and can also inhibit acetylcholine release from thalamic projecting neurons. Ketamine binds to inhibitory interneurons in the cerebral cortex (e.g., medial prefrontal cortex, insular cortex), limbic system, and hippocampus, leading to uncoordinated neuronal excitation and the emergence of an active electroencephalogram pattern, and subsequent loss of consciousness [137, 138]. Analgesic and sedative drugs play major roles in the prevention and treatment of specific systemic complications of ABI, such as PSH [6] and NPE [135]. Moreover, in severe TBI cases treated according to the Lund Concept, patients receiving analgesics and sedatives demonstrate remarkably low rates of systemic complications [142, 143].

Receptor modulators also hold promise in the clinical management of NODS. β-blockers and α-agonists have potential clinical applications for improving outcomes following ABI [144–146]. Although β-blockers have been shown to reduce mortality in TBI, their efficacy remains incompletely established [147]. Due to the lack of high-quality evidence, the use of this medication in TBI is restricted, underscoring the need for further validation of its actual efficacy through high-quality randomized trials [148]. These medications, by targeting specific receptor pathways, offer an alternative approach to managing the systemic dysregulation associated with NODS.

Neuromodulation methods, such as vagus nerve stimulation and sympathetic ganglion blockers, have emerged as innovative clinical interventions. Although noninvasive, their application requires careful ethical evaluation, including consideration of organ dysfunction caused by excessive vagal inhibition or sympathetic hyperactivity, or systemic instability. These approaches directly modulate the activity of key neural circuits involved in maintaining systemic homeostasis, offering a non-pharmacological strategy for the treatment of NODS [149, 150]. Spatially, these interventions can target multiple levels, including neuronal excitability in the brain, afferent and efferent nerves, and peripheral effector receptor regulation. Temporally, early-stage therapy may involve the combined use of analgesic and sedative drugs, followed in later stages by targeted receptor modulation and neuromodulation of affected effectors.

Graph-theoretic analysis has been employed to evaluate the functional and structural connectivity changes in specific brain networks following ABI [151–153]. The small-world properties of these networks can also be quantified [154–156]. The balance and dynamics of segregation and integration of brain functions can be tracked using time-resolved functional connectivity derived from resting-state functional magnetic resonance imaging data [157]. Although bedside magnetic resonance imaging has been used to assess brain injury in patients with ABI, significant challenges remain in conducting CSS analysis using functional magnetic resonance imaging in patients with NODS following ABI. These challenges include logistical support issues for patients, interference from intracranial metal implants, uncertainty regarding the clinical benefits of functional magnetic resonance imaging analyses, and ethical considerations concerning whether patients are likely to benefit from the analysis. Furthermore, there is currently a lack of clinical cases, and substantial relevant research is needed to validate the conceptual theory we have proposed. The absence of diagnostic biomarkers for NODS constitutes a barrier that hinders the translation of theoretical concepts into practical clinical applications. In addition, ensuring that medical professionals possess a comprehensive understanding and acceptance of this novel concept is crucial, as resistance or misunderstanding may impede its adoption and implementation. If network analysis studies based on CAS or CSS in patients with NODS following ABI can be realized, they would open a window for us to explore the central neurogenic mechanisms underlying the survival system. Autonomic function assessment is already clinically feasible, with metrics such as heart rate variability measurable at the bedside using non-invasive tools. Patients can also undergo real-time tracking of systemic parameters, such as blood pressure and temperature. Comprehensive analysis can also include intracranial pressure, cerebral oxygenation, and blood gas analyses.

As CAS and CSS are also key parts of the survival system in the human body, their pathology or injury usually indicates a state of critical illness. Therefore, the challenges of using a model of NODS following ABI to study CAS or CSS connectivity are as follows: 1) the subjects require neurointensive care; 2) complementary peripheral markers must be developed to facilitate the study of the organization and connectivity of these neural components. Certain bedside continuous monitoring variables, such as heart rate variability and baroreflex sensitivity, represent ANS output and have the potential to assess autonomic dysfunction. When combined with other continuously monitored homeostatic variables, these metrics can not only provide individualized evaluation of systemic stability and serve as treatment targets, but also function as complementary tools for decoding and mapping the connectivity of the control center [158, 159].

## Conclusions

In conclusion, NODS following ABI reflect systemic instability resulting from central network dysfunction and may represent an important factor affecting the outcomes of ABI patients. In clinical practice, the possible neuropathophysiological mechanisms of NODS can be interpreted from the perspective of complex brain network theory, encompassing both structural and functional networks characterized by small-world networks, hierarchy, hubs, and modularity. The integration of these networks supports an efficient, large-scale brain system and ensures the competitive optimization of information segregation and integration.

## Supplementary Information

**Additional file 1.** **Table S1** Clinical investigation of systemic manifestations after ABI.

## Data Availability

Not applicable.

## Abbreviations

ABI: Acute brain injury
ANS: Autonomic nervous system
CAN: Cortical autonomic network
CAS: Central autonomic system
CNS: Central nervous system
CSS: Central stress system
CSW: Cerebral salt wasting
Hippo: Hippocampus
ICU: Intensive care unit
LC: Locus coeruleus
MODS: Multiple organ dysfunction syndrome
NODS: Neurogenic organ dysfunction syndrome
NPE: Neurogenic pulmonary edema
NTS: Nuclei of the tractus solitarius
PAG: Periaqueductal gray
PSH: Paroxysmal sympathetic hyperexcitation
PVN: Paraventricular nucleus of the hypothalamus
SAN: Subcortical autonomic network
SNS: Sympathetic nervous system
TBI: Traumatic brain injury
