# Supplementary material for: Neurogenic organ dysfunction syndrome after acute brain injury
Source: Mil Med Res. 2025 Nov 7;12:77. doi: 10.1186/s40779-025-00662-8 (PMC12593802; doi:10.1186/s40779-025-00662-8)
Supplement: Supplementary file 1 — Additional file 1. Table S1 Clinical investigation of systemic manifestations after ABI. [file 40779_2025_662_MOESM1_ESM.pdf]

Table S1 Clinical investigation of systemic manifestations after ABI

Reference	Study design	Type of brain injury	Number of patients	Frequency of system complications									Associated with worse outcome
				Cardiovascular	Respiratory	Digestive	Immunological	Metabolic	Haematological	Electrolytical	Infectious	Other	
Piek et al. [1]	Retrospective cohort	TBI	734	29.3%	40.6%	Rarely	NA	NA	18.4%	59.3%	Sepsis (10%)	Liver, or kidney failure rarely	Yes
Solenski et al. [2]	Prospective cohort	SAH	457	NA	PE (23%)	NA	NA	NA	Thrombocytopeni (4%)	EI (33%)	NA	Liver dysfunction (24%)<br>Kidney dysfunction (7%)	Yes
Yoshimoto et al. [3]	Retrospective cohort	SAH	103	1.9%	11.7%	NA	SIRS (54%)	Hyperglycaemia (65%)	2.9%	NA	NA	Kidney failure rarely	Yes
Zygun et al. [4]	Retrospective cohort	TBI, SAH	55	82%	80%	NA	NA	NA	5.5%	NA	NA	No liver, or kidney failure rarely	No
Satoh et al. [5]	Retrospective cohort	SAH	1431	CF (2.6%)<br>VT/VF (1.8%)<br>ST/T change (33.6%)	NPE (14.6%)<br>Apnea (10.9%)	NA	NA	NA	NA	NA	NA	NA	Yes
Zygun et al. [6]	Prospective cohort	TBI	209	52%	81%	NA	NA	NA	36%	NA	NA	Liver dysfunction (8%)<br>Kidney dysfunction (7%)	Yes
Mikalsen et al. [7]	Retrospective cohort	TBI	133	Hypotension (20%)	Hyoxemia (10%)<br>Pneumonia (71%)<br>ARDS/ALI (26%)<br>Hypercapnia (25%)	NA	NA	Hyperglycaemia (26%)<br>Hypoalbuminemia (31%)	Anaemia (22%)<br>Coagulation disorder (6%)	Hyponatremia (10%)	Sepsis (6%)	Liver, or kidney failure rarely	Yes
Mascia et al. [8]	Prospective cohort	TBI, CA, BT	373	NA	Respiratory failure (48%)	NA	NA	NA	NA	NA	Sepsis (16.4%)<br>Septic Shock (11.5%)	Liver failure (2.4%)<br>Kidney failure (24.7%)	Yes
Alberti et al. [9]	Prospective cohort	Stroke	1101	VTE (7.8%)<br>Myocardial infarction (3%)	PE (1.4%)<br>Pneumonia (9.1%)	NA	NA	NA	DVT (7.5%)	NA	Urinary Infections (10.4%)	NA	Yes
Corral et al. [10]	Retrospective cohort	TBI	224	Arrhythmias (11%)<br>Hypotension (44%)<br>Hypertension (12%)	ARDS (9%)<br>Atelectasis (21%)<br>$PaO_2/FiO_2 < 200$ (41%)<br>PaO_2/FiO_2 (200 – 300) (42%)	18%	NA	NA	27%	21%	Sepsis (75%)<br>Septic shock (6%)<br>Respiratory infections (68%)	AKI (8.0%)	Yes
Omar et al. [11]	Retrospective cohort	TBI	12887	MI (0.8%)<br>Cardiac arrest (0.5%)	PE (1%)<br>AP (1.9%)<br>HAP (10.4%)<br>ARDS (1.4%)	CDC (1.3%)	NA	NA	DVT (3.5%)<br>Coagulopathy (0.6%)	NA	WI (1.4%)<br>Sepsis (2.2%)<br>CVCRI (0.2%)	AKI (2.0%)<br>Delirium (4.8%)	Yes
Goyal et al. [12]	Prospective cohort	TBI	154	34.4%	61.0%	Ileus (1.3%)<br>Diarrhea (13.0%)	NA	Bilirubin (2 mg/dl) (1.9%)	33.1%	46.1%	Sepsis (20.1%)<br>Septic shock (5.8%)	AKI (3.9%)<br>Increase AST (1.3%)	Yes
Astarabadi et al. [13]	Retrospective cohort	TBI	285	12%	23%	NA	NA	NA	4%	NA	NA	Liver (8%)<br>Kidney (3%)	Yes

ABI acute brain injury, *TBI* traumatic brain injury, *NA* not available, *SAH* subarachnoid hemorrhage, *PE* pulmonary embolism, *EI* electrolyte imbalance, *SIRS* systemic inflammatory response syndrome, *CF* cardiac failure, *VT/VF* ventricular tachycardia or fibrillation, *NPE* neurogenic pulmonary edema, *ARDS* adult respiratory distress syndrome, *ALI* acute lung injury, *CA* cerebrovascular accident, *BT* brain tumor, *VTE* venous thromboembolism, *DVT* deep venous thrombosis, *PaO2/FiO2* arterial oxygen pressure/oxygen inspired fraction ratio, *AKI* acute kidney injury, *MI* Myocardial infarctus, *AP* aspiration pneumonia, *HAP* hospital-acquired pneumonia, *CDC* clostridium difficile colitis, *WI* wound infection, *CVCRI* central venous catheter-related infection, *AST* aspartate aminotransferase

Reference

1. Piek J, Chesnut RM, Marshall LF, van Berkum-Clark M, Klauber MR, Blunt BA, et al. Extracranial complications of severe head injury. J Neurosurg. 1992;77(6):901-7.
2. Solenski NJ, Haley EC, Jr., Kassell NF, Kongable G, Germanson T, Truskowski L, et al. Medical complications of aneurysmal subarachnoid hemorrhage: a report of the multicenter, cooperative aneurysm study. Participants of the Multicenter Cooperative Aneurysm Study. Crit Care Med. 1995;23(6):1007-17.
3. Yoshimoto Y, Tanaka Y, Hoya K. Acute systemic inflammatory response syndrome in subarachnoid hemorrhage. Stroke. 2001;32(9):1989-93.
4. Zygun DA, Doig CJ, Gupta AK, Whiting G, Nicholas C, Shepherd E, et al. Non-neurological organ dysfunction in neurocritical care. J Crit Care. 2003;18(4):238-44.
5. Satoh A, Nakamura H, Kobayashi S, Miyata A, Matsutani M. Management of severe subarachnoid hemorrhage; significance of assessment of both neurological and systemic insults at acute stage. Acta Neurochir Suppl. 2005;94:59-63.
6. Zygun DA, Kortbeek JB, Fick GH, Laupland KB, Doig CJ. Non-neurologic organ dysfunction in severe traumatic brain injury. Crit Care Med. 2005;33(3):654-60.
7. Schirmer-Mikalsen K, Vik A, Gisvold SE, Skandsen T, Hynne H, Klepstad P. Severe head injury: Control of physiological variables, organ failure and complications in the intensive care unit. Acta Anaesthesiol Scand. 2007;51(9):1194-201.
8. Mascia L, Sakr Y, Pasero D, Payen D, Reinhart K, Vincent JL. Extracranial complications in patients with acute brain injury: a post-hoc analysis of the SOAP study. Intensive Care Med. 2008;34(4):720-7.
9. Alberti A, Agnelli G, Caso V, Venti M, Acciarresi M, D'Amore C, et al. Non-neurological complications of acute stroke: frequency and influence on clinical outcome. Intern Emerg Med. 2011;6 (Suppl 1):119-23.
10. Corral L, Javierre CF, Ventura JL, Marcos P, Herrero JI, Mañez R. Impact of non-neurological complications in severe traumatic brain injury outcome. Crit Care. 2012;16(2):R44.
11. Omar M, Moore L, Lauzier F, Tardif PA, Dufresne P, Boutin A, et al. Complications following hospital admission for traumatic brain injury: a multicenter cohort study. J Crit Care. 2017;41:1-8.
12. Goyal K, Hazarika A, Khandelwal A, Sokhal N, Bindra A, Kumar N, et al. Non- neurological complications after traumatic brain injury: a prospective observational study. Indian J Crit Care Med. 2018;22(9):632-8.
13. Astarabadi M, Khurrum M, Asmar S, Bible L, Chehab M, Castanon L, et al. The impact of non-neurological organ dysfunction on outcomes in severe isolated traumatic brain injury. J Trauma Acute Care Surg. 2020;89(2):405-10.
